# Head and Neck Restoration in Scar Alopecia: Hair Transplantation in Scalp, Eyebrows, Beard and Mustache

**DOI:** 10.29252/wjps.10.3.90

**Published:** 2021-09

**Authors:** Ahmad Noori, Mina Rabiee, Davood Mehrabani, Mohammad Reza Namazi

**Affiliations:** 1 Department of Hair Transplantation, Novin Iran Clinic, Shiraz, Iran; 2 Department of Genetics, Islamic Azad University, Shahrekord Branch, Shahrekord, Iran; 3 Burn and Wound Healing Research Center, Shiraz University of Medical Sciences, Shiraz, Iran; 4 Stem Cell Technology Research Center, Shiraz University of Medical Sciences, Shiraz, Iran; 5 Comparative and Experimental Center, Shiraz University of Medical Sciences, Shiraz, Iran; 6 Li Ka Shing Center for Health Research and Innovation, University of Alberta, Edmonton, AB, Canada; 7 Research Center, Shiraz University of Medical Sciences, Shiraz, Iran

**Keywords:** Scar alopecia, Hair transplantation, Scalp, Eyebrow, Beard, Mustache

## Abstract

**BACKGROUND:**

Surgical management of hair loss has become an increasingly challenging procedure, when dealing with scar alopecia. We investigated the efficacy of hair transplantation in patients with head and neck scar alopecia.

**METHODS:**

From 2016 to 2018 in Shiraz, Iran, all patients with scar alopecia in head and neck were evaluated for efficacy of follicular unit extraction (FUE), follicular unit transplantation (FUT) or a combination of two methods from donor sites in scalp and beard various hair-grafts were compared.

**RESULTS:**

Fifty-six patients were enrolled. Most of them were between 31 and 40 yr old (48.3%) and male (71.4%). Trauma, burn, surgical excision of adjacent skin, radiotherapy and leishmaniasis were the registered causes. Scars were visible in scalp (39.3%), beard (28.6%), eyebrow (21.4%), and moustache (10.7%) regions. FUE (87.5%), FUT (10.7%) and a combination (1.8%) were the used methods. One-hair-grafts were used in eyebrows (100%), moustache (100%), beard (88%) and scalp (7.9%), while 2-hair-grafts in beard (6%) and scalp (47.4%) and 3-haired grafts in beard (6%) and scalp (44.7%) transplantations.

**CONCLUSION:**

In head and neck scar alopecia, hair transplantation was selected based on type and depth of scar. FUE was targeted when huge grafts were not needed, including beard, moustache, and eyebrow, while FIT was used when extensive scars were present in scalp. One-hair-grafts were mostly applied for eyebrow, moustache and beard, 2-hair- and 3-hair- grafts for beard and scalp transplantation. These findings can be added to the literature when FUE, FIT, or their combination are targeted in hair restoration of scar alopecia in head and neck.

## INTRODUCTION

Hair loss is still a significant problem worldwide resulted into enrolling more than 600,000 surgical hair restoration procedures in 2016^1^. Hair loss can happen temporary or permanent and can be local or diffuse due to physiologic, as noted in normal aging, or iatrogenic as observed in burns, trauma, chronic diseases, iron deficiency, thyroid disorders, high fever, consuming some medications, chemo- and radiotherapy, exposure to chemicals, hormonal changes, androgenetic factors, and drastic diets^2^. Hair loss can occur due to generalized or local skin diseases, such as Lichen planopilaris, lupus, and alopecia areata^2^. Stress was shown to accelerate a genetically programmed hair loss^3^.

Scarring alopecia, named as cicatricial alopecia is an irreversible inflammatory follicular damage associated with hair loss^4^. As a result, surgical management of scarring alopecia has become an increasingly popular and satisfying procedure for both men and women and this is a more challenge when treating patients with burn scarring alopecia^5^. Hair transplantation has been conducted for decades and has experienced a cosmetic revolution in the 21^st^ century and identical to all surgical procedures, there has been a continual evolution in this procedure with the goal of an ever safer, efficient, and even higher level of patient satisfaction^1^. In the earliest hair transplants, a punch was used to extract donor hair follicles for repair of regions of the moustache, beard, and eyebrows scarred by burns^6^, but recently, surgical management of hair loss has become an increasingly satisfying and popular method^7^. 

A successful hair transplant procedure should consider the extent of hair loss, density of donor hair, and caliber and color of hair, and also a realistic expectation of the patient and physician. A challenge for the hair transplant surgeon is to reach an acceptable distribution of transplanted hair and creates a permanent natural hairline and over time, the transplanted hair remains cosmetically satisfying for the patient and physician^7^. Consultation is the key point for a successful procedure considering the key points in every consultation including the history of hair loss, the patient’s physical examination, the patient’s goals, the use of medications in treatment of the hair loss, and where to transplant. The key points in the patient’s physical examination are the donor density (hair follicles per cm^2^), the caliber of the patient’s hair, and the extent of hair loss^8^. 

The harvesting of donor hair follicles can be accomplished by amputating a strip of hair-baring skin called strip harvesting or follicular unit transplantation (FUT) method and by removing each follicle individually via follicular unit extraction (FUE)^9^. FUE was described as a method of harvesting follicular units from a donor site and the surgeon extracts individual follicular units with circular punches (0.8 to 1 mm in diameter) that heal by second intention within a few days^10^. 

It is a procedure that makes transplantation surgery less invasive with no need to sutures, with no linear scar and results in a very high percentage of transected follicles. It is a very slow technique as the follicular grafts are extracted individually from various body sources such as scalp and beard^11^. FUE is a procedure that allows to pick desirable follicular units individually without any need to suturing and with minimal scarring^12^. The procedure involves making a submillimeter punch incision surrounding a single follicular unit extending into the dermis using a motorized sharp punch of 0.9 or 1.0 mm in diameter^10^. The follicles are then extracted individually by a pair of fine forceps, leaving an indiscernible donor scar^10^. 

In a majority of patients, the donor hairs harvested by FUE technique avoids a linear donor site incision and patients typically can cut the hair as short as desired. FUT is another harvesting technique via the excision of a small elliptical strip of skin, usually from the occipital scalp, also called strip harvesting^13^, but the main disadvantage of FUT is formation of a sizable donor scar that can be an issue for patients who prefer shorter hairstyles^10^. In FUT, surgical removal of a strip of hair-bearing scalp tissue is usually 0.6-1.0 cm wide and 8-20 cm long. Traditionally the donor hair is divided into grafts containing 10-25 hairs each^14^. 

Burn injuries, whether caused by flame, scalds, chemicals and even electrical injury can affect a large body surface area and can be isolated to the head and neck region leaving severe scarring which is hard to hide with clothing, specifically the scalp, beard region, eyebrows, and eyelashes that makes hair transplantation more challenging^15^. As hair loss due to scarring can have a great impact on the self-esteem and quality of life of patients than the scars themselves and act as a constant reminder of the causative traumatic incident to the patient, the efficacy of FUE, FUT or a combination of two methods from donor sites in scalp and beard was assessed in head and neck scarring alopecia.

## MATERIALS AND METHODS

From Mar 2016 to Mar 2018, all patients from both sexes and various age groups who referred to Novin Iran Clinic in Shiraz, Iran and suffered from scarring alopecia in scalp, eyebrows, moustache and beard due to burn, surgical excision of adjacent skin, trauma, radiotherapy and leishmaniasis were enrolled. Those with a history of skin diseases in active phase such as alopecia areata, Lichen planopilaris were excluded from the study. Treatment plans were made after a thorough history taking of any relevant medical condition or medication, patient-doctor consultation results, physical examination and type and depth of scars. 

Once a patient scheduled the procedure, preoperative and postoperative guidelines were shared with the patient and a written signed consent letter was provided. An ethical approval was also received from institute Ethics Committee based on Declaration of Helsinki. 

All patients could contact us with any questions regarding the procedure protocol. During consultation, the type and depth of scars and the proper caliber and density of the hair in the donor region were evaluated. Before treatment, we had an appointment with the patient in front of a mirror with a grease pencil or marker to draw in the area of planned hair placement to reach an agreement on a realistic place for hair placement and if FUT was needed, the optimal donor region was defined.

During the procedure, the patient was asked to be in the prone or supine position to allow us a better visibility and stability for donor harvesting and to reduce the incidence of vasovagal episodes during donor harvesting. The patient was premedicated to help relaxation and bearing the hours of surgery much more easily with an oral anxiolytic medicine such as diazepam (10-20 mg), 30 min before the surgery. Patients underwent hair restoration using FUE, FUT or a combination of two methods from donor sites in the scalp and beard based on the type and depth of scars. 

For all patients if FUT was necessary, the protocol was explained to be an elliptical donor strip provided from posterior scalp. For FUE technique, transplanted hair avoided a linear donor site incision and patients typically could cut the hair as short as desired. The transplanted hair would begin to grow 4 months after the procedure and improve gradually until 12 months^16^. During the procedure, patients were comfortable and could listen to music, watch a movie, or relax. They could inform us if they were feeling any discomfort to administer more local anesthetics. Patients were instructed to expect a probable swelling along the forehead and eyes for 3 to 5 d after starting the procedure. They were visited in the clinic on the 1^st^ postoperative day to learn how to wash the hair and to take care of the grafts without allowing the shower water to directly hit the grafts until one week. The crusts were gently removed after a week.

In FUE procedure, a submillimeter punch incision was created surrounding a single follicular unit extended into the dermis. Technique and operator standardization were carried out using a specific protocol. Local anesthesia was conducted for all patients using 1% lidocaine together with 1:100,000 epinephrine. Two registered nurses trained by the first author were responsible for FUE and each patient was randomly enrolled by one of the nurses. The punches varied in size ranging from 0.75 to 1.2 mm in diameter attached to a motorized sharp punch (Folligraft; LeadM Company, Seoul, Korea). The follicles were extracted individually by a pair of fine forceps. 

The average procedure in each session took 6 hours. The donor areas were the occiput or the parietal scalp or the beard. Regarding restoration of beard, moustache and eyebrow, all patients received the grafts via FUE technique. One-hair-grafts were mostly applied for the eyebrow, moustache, and beard; whereas 2-hair-grafts for beard and scalp and 3-hair-grafts just for the scalp and beard. When the hairs within a graft were divergent in their growth, 2-hair grafts were not used. 

After the procedure, patients returned home with a pressure dressing that remained on overnight. They received prednisone (30 mg/d for 3 d) to minimize frontal edema and acetaminophen (2-4 tablets) for pain relief. Patients applied repair creams to the donor site twice daily for 1 week after surgery. Topical antibiotics were administered to the donor area twice daily for a week. They could take shower daily to allow the water to rinse off the scalp, but they avoided scratching or scrubbing at the scalp. The perifollicular crusting around the grafts was removed after one week and shaving was allowed after 2 weeks. Patients could have their make up in the eyebrow area one week post-operation after all the crusts were fallen out.

The FUT procedure was done under local anesthesia using 1% lidocaine together with 1:100,000 epinephrine to minimize the bleeding. When the incisions were made, 10 to 30 mL of saline was administered to produce turgor in the donor ellipse and if patients were anxious about pain, they underwent monitored sedation. The procedure was divided into 4 steps of (i) Extraction of the donor strip, (ii) Microscopic dissection of the donor strip into follicular units, (iii) Creation of recipient incisions and (iv) Implantation of follicular grafts. 

In FUT, the region between the occipital protuberance and 2 cm above the top of the ears was the selected region for harvesting the hair as a band of about 1.5-2.5 cm wide and 30 cm long using a scalpel blade of number 10. The incision was undertaken clean parallel to the line of the hair follicles and at a depth of 5 to 7 mm below the bulbs of the follicles to reach minimal transection of hair follicles. To have optimal visibility, skin hooks on both sides of the ellipse were used for retraction. The ellipse was removed with scalpel or scissors. If needed, hemostasis was conducted by electrocautery. 

For graft site creation, various instruments were used including 19- and 20-gauge needles (South Korea), chisel and custom made blades to make sites as small as 0.6 and 0.7 mm in diameter. The scalpel blades used for 1-hair-grafts were 0.6 to 0.7 mm, while those used for grafts containing 2-hairs-, and 3-hairs-grafts were 0.9, and 1.0 mm. The strip was divided in slivers, and then, each sliver was divided into individual FUs of 1-, 2-, 3-, or 4-hair grafts depending on how they existed in the scalp. The donor region was repaired with sutures in a single plane with 4-0 nylon joining the edges carefully and precisely. Once the last suture was carried out to close the donor site, a temporary dressing was applied, and the patient turned over again. The sutures were removed after a week. To achieve acceptable density, we implanted between 20 and 40 units/cm^2^ based on blood supply in the area of scar. The patient’s hairline design was shown in front of a mirror.

The graft placement involved two specialized nurses to move the hair efficiently into the graft sites using forceps with curved tips, straight tips, cotton-tipped applicator, and Choi implanter under good ergonomics and lighting. The grafts were continuously well hydrated in physiological saline solution throughout the entire process from extraction to implantation to improve the survival rate. In insertion of the grafts into the recipient incisions, the two registered and trained nurses worked together, 1 on either side of the patient, introducing the follicular grafts by straight or angled fine-tipped jeweler’s forceps and hair implanters into the incisions individually inserting about 10 units/min. 

Once the grafting was finalized, an overnight dressing was applied, and patients could return home. They were instructed to be cautious not to traumatize the newly placed grafts. The detailed timeline for expected hair growth were shared with the patient and were instructed for the medications including antibiotics, minoxidil, supplements such as zinc, 5% Rogaine (5% minoxidil, Johnson & Johnson Consumer Inc., USA) and platelet rich plasma (PRP) therapy two times per year. The patient was asked to be visited the following day. The frequency of further visits was dependent on the patient’s status and the presence of any probable complications that was weekly during the first month after the procedure followed by a 6-month interval.

## RESULTS

Fifty-six patients were enrolled in this study. Most of them were 31-40 yr old (48.3%), and the least were older than 50 yr old age groups (3.5%, age range of 21-60 yr old). Male subjects consisted 71.4% of the patients. Trauma (55.4%) was the most causative agent responsible for scar alopecia and the least were radiotherapy (1.8%) and leishmaniasis (1.8%) registered in their medical history leaving scar mostly in in scalp (39.3%), and the least in moustache (10.7%) regions (Table 1).

The used surgical designs were FUE (87.5%), FUT (10.7%) and a combination of both surgical procedures (1.8%). When FUE was targeted for transplantation, scalp (83.7%) and beard (16.3%) were the harvesting donor regions. For scalp, the maximum number of grafts (1314, range: 150-3360) was used and the least grafts in moustache (80, range: 40-207, Table 1). The 2-hair-grafts were the most possible used grafts for transplantation (mean=772, range: 100-1800), followed by 1-hair-grafts as the least used grafts (mean=220, range: 50-400, Table 1). 

One-hair-grafts were used mostly in in eyebrows and moustache (100%), while 2-hair- and 3-hair-grafts mostly in scalp transplantations (47.4%, 44.7%, respectively, Table 1). Within 4 d after a normal washing, the perifollicular crusts started shedding. The inflammation in transplanted follicles by FUT resolved after 2 wk and the transplanted hairs started to grow after 4 months. We noticed a follicular mean survival percentage of greater than 75 percent for the enrolled patients.

**Fig. 1:**
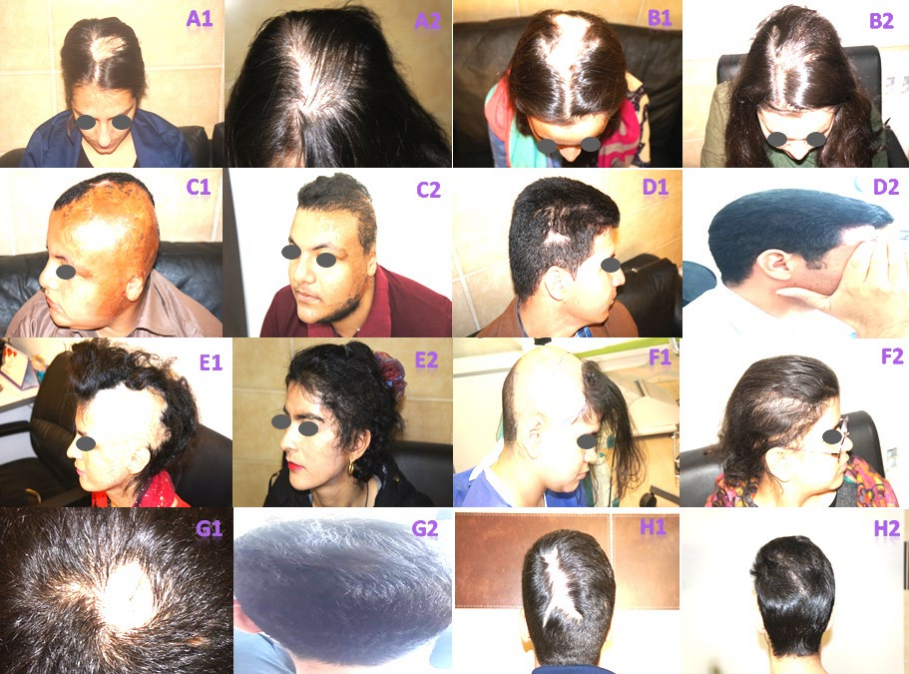
Causes and number of hair grafts in scarring lesions of the scalp before and after transplantation. **A:** FUE of 1320 two-hair, and 700 three-hair grafts post-traumatic scarring alopecia. **B:** FUE of 700 two-hair, and 400 three-hair grafts in Kerion scarring alopecia. **C: **FUE of 360 one-hair, 950 two-hair, and 1015 three-hair grafts in burn scarring alopecia. **D:** FUE of 135 two-hair, and 135 three-hair grafts post-traumatic scarring alopecia. **E:** FUE of 200 one-hair, 1800 two-hair, and 1000 three-hair grafts in burn scarring alopecia. **F: **FUE and FUT of 1460 two-hair, and 1900 three-hair grafts post-radiotherapy scarring alopecia. **G:** FUE of 300 three-hair grafts in Morphea scarring alopecia. **H:** FUE of 270 two-hair, and 730 three-hair grafts in Kerion scarring alopecia

**Fig. 2:**
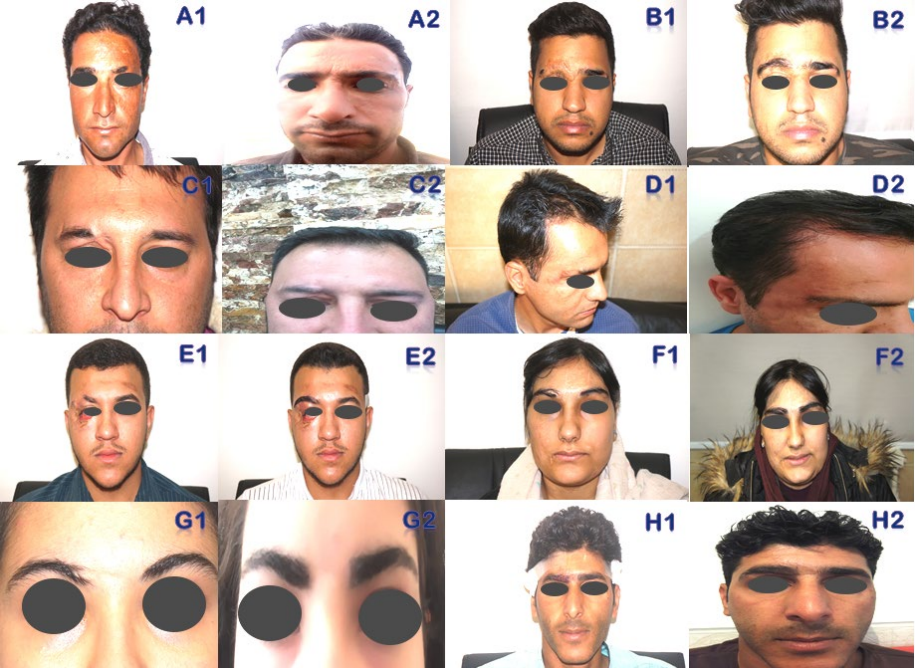
Causes and number of hair grafts in scarring lesions of the beard and moustache before and after transplantation. **A: **FUE of 1850 one-hair grafts in burn scarring alopecia. **B:** FUE of 420 one-hair grafts in leishmaniasis scarring alopecia. **C:** FUE of 1150 one-hair, and 900 two-hair grafts in burn scarring alopecia. **D:** FUE of 1150 one-hair, and 900 two-hair grafts in burn scarring alopecia. **E:** FUE of 400 one-hair, and 750 two-hair grafts in burn scarring alopecia. **F:** FUE of 1150 one-hair, and 900 two-hair grafts post-traumatic scarring alopecia. **G:** FUE of 150 one-hair grafts using beard source in burn scarring alopecia. **H:** FUE of 120 one-hair grafts using beard source post-traumatic scarring alopecia

**Fig. 3:**
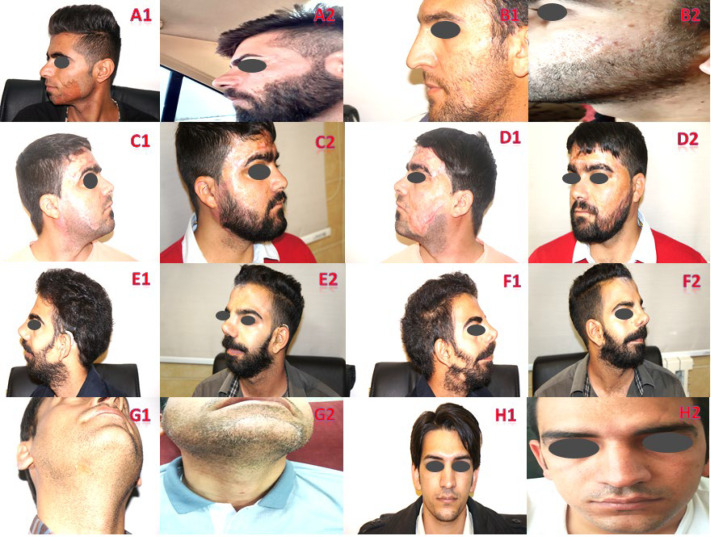
Causes and number of hair grafts in scarring lesions of the eyebrows before and after transplantation. **A:** FUE of 180 one-hair grafts in burn scarring alopecia. **B:** FUT of 260 one-hair grafts post-traumatic scarring alopecia. **C:** FUE of 170 one-hair grafts in post-traumatic scarring alopecia. **D:** FUE of 120 one-hair grafts in post-traumatic scarring alopecia. **E:** FUT of 506 one-hair grafts in post-traumatic scarring alopecia. **F:** FUE of 321 one-hair grafts in post-traumatic scarring alopecia. **G:** FUE of 220 one-hair grafts in post-traumatic scarring alopecia. **H:** FUE of 451 one-hair grafts in post-traumatic scarring alopecia

Figure 1 depicts the causes and the number of hair grafts in scarring lesions of the scalp using one-hair-, two-hair-, and three-hair-grafts in burns, Morphea, Kerion, post-traumatic, and post-radiotherapy scarring alopecia using FUE and FUT methods. Figure 2 illustrates the causes and number of hair grafts in scarring lesions of the beard and moustache using one-hair- and two-hair-grafts in burn, leishmaniasis and post-traumatic scarring alopecia via FUE method. Figure 3 demonstrates the causes and number of hair grafts in scarring lesions of the eyebrows using one-hair-grafts in burn and post-traumatic scarring alopecia by FUE and FUT methods.

## DISCUSSION

Scarring alopecia from secondary causes such as burn, surgery, and trauma are mostly amenable to hair transplantation^2^. In our study, trauma, burns, surgical excision of adjacent skin, radiotherapy and leishmaniasis were the major causes in the medical history leaving scar in scalp, beard, eyebrow, and moustache regions. Nowadays, restoration of scalp, eyebrows, beards, and mustache have all become popular procedures when placed properly in a natural direction, angle, and pattern with outstanding results benefiting from hair transplants^17^. 

Similar to our study, FUE was the mostly selected procedure for patients with scar alopecia^17^. FUE is supported as a minimally invasive procedure to obtain follicular units, and it lacks a long linear scar that the patient already suffers from, and does not need sutures and results in a better wound healing^18^. In hair transplantation, both techniques of FUE and FUT can be state of the art with advantages and disadvantages for the patient considered during a hair transplant consult. Therefore, someone who likes to wear his hair short, FUE should be chosen as the preferred harvesting technique because scarring from FUE is less visible than a linear scar from FUT^18^. Meanwhile, women do not usually wish to trim hair to 1 mm in length necessary for FUE, which can make elliptical donor harvesting the other choice when needed^1^.

As our findings showed, graft counts played an important role in restoration of scarring alopecia. Epstein reported graft counts for the moustache area to range from 300 to as many as 850 grafts, and 350 to as many as 500 grafts per cheek beard^17^. In our study for scalp, 150-3360 grafts were inserted and the ranges for beard, eyebrow and moustache were 20-2050, 75-700 and 40-207 grafts, respectively that denotes to a higher graft counts from scalp and beard as available harvesting donor regions. The difference is dependent on how much if any preexisting hairs were present, the thickness of the donor hairs, and of course the exact desired shape and density of hairs and the type and depth of scars^19^.

In eyebrow transplantation, mostly 1-hair-grafts were used, because natural pattern of human eyebrow hairs happens in singular follicular units^20^. In our study, similarly one-hair-grafts were used for all eyebrows transplants and incisions were created at an angle as acute as possible to the skin, because within the medial-most part of the eyebrow inferiorly, the hairs grow in a superior and lateral direction. While moving much more superior within the head of the eyebrow, the direction was altered to a more lateral and inferior direction. Significant variations were considered regarding the patient’s gender during hair transplantation.

The recommendation for hair transplantation over the moustache and beard area is to place 1-hair- to 2-hair-grafts^21^. In our study in beard, 1-hair-, 2-hair- and 3-hair-grafts included 88%, 6% and 6% of the total grafts, respectively. The majority of our patients were 31-40 yr old. We did not notice age to be a determining factor and the result were satisfactory in all age groups. Similar findings were reported by other researchers in candidate selection, while age was not a determining factor too^1^. Women have been a minority among patients undergoing hair transplantation for scarring alopecia too^20^; and in our study, male and female subjects consisted 71.4% and 28.6% of the patients, respectively denoting to the minority of women undergoing hair transplantation.

Currently in hair transplant clinics, the majority of patients are recommended for medical therapy to maximize the efficacy of any transplant, to stimulate hair growth and to enhance graft survival including minoxidil and finasteride^1^, low level laser light therapy^22^, and platelet-rich plasma (PRP) as treatment options^23^^,^^24^. Successful outcomes have been reported with the use of tissue engineered dermal regeneration templates followed by follicular unit transplantation to reconstruct large scalp defects^25^^,^^26^. In our study, patients received minoxidil and if patients were willing, PRP was added as complementary treatment options.

There could be some limitations in all studies using hair transplantation in scarring alopecia including complications encountered in hair transplantation surgery such as enlarged scars, folliculitis, keloid, necrosis neuralgias, donor hair effluvium, and arteriovenous fistulas in the donor area^27^. Moreover, there are significant challenges such as limited donor supply, staff training, the length of time of the procedure, and developing new medications^8^^,^^28^. Our center was rich in technical skills and enrolled artistic abilities to create an appropriate site angling and hairline design that fitted with the patient’s overall features^29^. In our patients, there was the challenge of limited donor supply in scalp of some patients that was effectively compensated in male subjects by use of non-scalp body hair such as beard as potential sources of donor grafts. As hairs grow slightly laterally and then downward along the goatee, it is of great importance to make an angle as acute as possible to the skin^16^. The creation of density in this area was a little difficult in our center due to the topography of the upper lip’s cupid’s bow area. Therefore, a maximal density packing of 2-hair-grafts were used. When approaching eyebrow restoration, it is important to monitor the shape of masculine eyebrow versus feminine eyebrow appearance. As the masculine eyebrow shape is generally less arched, it comes to a lateral widening at the peak of the brow, whereas the feminine eyebrow shape is more rounded and arched and we tried to cover the patients’ expectations. No complication happened in our patients and they were all satisfied with the results. 

**Table 1:** Hair restoration in head and neck scar alopecia

Variable		Amount
Cause	Trauma	55.4%
	Burn	28.5%
	Surgical excision of adjacent skin	12.5%
	Radiotherapy	1.8%
	Leishmaniasis	1.8%
Sex	Male	71.4%
	Female	28.6%
Age (yr)	21-30	26.8%
	31-40	48.3%
	41-50	21.4%
	>50	3.5%
Scar region	Scalp	39.3%
	Beard	28.6%
	Eyebrow	21.4%
	Moustache	10.7%
Surgical design	FUE	87.5%
	FUT	10.7%
	FUE+FUT	1.8%
Harvesting donor regions	Scalp	83.7%
	Beard	16.3%
Number of grafts (Range)	Scalp	1314 (150-3360)
	Beard	568 (20-2050)
	Eyebrow	290 (75-700)
	Moustache	80 (40-207)
Type of grafts (Mean, Range)	1-hair-grafts	(220, 50-400)
	2-hair-grafts	(772, 100-1800)
	3-hair-grafts	(750, 50-1900)

FUE: Follicular unit extraction, FUT: Follicular unit transplantation.

## CONCLUSION

Trauma and burn were demonstrated as the most prevalent causes of scarring alopecia in patients seeking for hair restoration and scar reduction. In head and neck scar alopecia, hair transplantation was selected based on type and depth of scar. FUE was targeted when huge grafts were not needed, including beard, moustache, and eyebrow, while FIT was used when extensive scars were present in scalp. One-hair-grafts were mostly applied for eyebrow, moustache and beard, 2-hair- and 3-hair- grafts for beard and scalp transplantation. These findings can be added to the literature when FUE, FIT, or their combination are targeted in hair restoration of scar alopecia in head and neck. 

## CONFLICT OF INTEREST

The authors declare that they have no conflict of interest and they present their consent to publish the article.
